# Home Range Size Variation in Female Arctic Grizzly Bears Relative to Reproductive Status and Resource Availability

**DOI:** 10.1371/journal.pone.0068130

**Published:** 2013-07-03

**Authors:** Mark A. Edwards, Andrew E. Derocher, John A. Nagy

**Affiliations:** 1 Royal Alberta Museum, Government of Alberta, Edmonton, Alberta, Canada; 2 Department of Biological Sciences, University of Alberta, Edmonton, Alberta, Canada; University of Manitoba, Canada

## Abstract

The area traversed in pursuit of resources defines the size of an animal’s home range. For females, the home range is presumed to be a function of forage availability. However, the presence of offspring may also influence home range size due to reduced mobility, increased nutritional need, and behavioral adaptations of mothers to increase offspring survival. Here, we examine the relationship between resource use and variation in home range size for female barren-ground grizzly bears (*Ursus arctos*) of the Mackenzie Delta region in Arctic Canada. We develop methods to test hypotheses of home range size that address selection of cover where cover heterogeneity is low, using generalized linear mixed-effects models and an information-theoretic approach. We found that the reproductive status of female grizzlies affected home range size but individually-based spatial availability of highly selected cover in spring and early summer was a stronger correlate. If these preferred covers in spring and early summer, a period of low resource availability for grizzly bears following den-emergence, were patchy and highly dispersed, females travelled farther regardless of the presence or absence of offspring. Increased movement to preferred covers, however, may result in greater risk to the individual or family.

## Introduction

A home range is defined as the area an animal traverses to acquire resources necessary to sustain the biological requirements of life [1]. Female home range size is expected to be a function of the spatial dispersion of forage [2]. Female reproductive status also affects home range size [3], because of the limited mobility of dependent young [4], the higher nutritional need associated with lactation [5], and the behavioral adaptations to increase offspring survival [6], [7]. Therefore, where female reproductive status and landscape characteristics differ, intraspecific variation in home range size is expected [8], [9], [10]. Spatial variation in seasonally available resources may also affect home range size.

Home range size may be predicated on an animal’s size-dependent metabolic rate and on the productivity of its habitat [11]. Both the abundance and spatial configuration of important covers within the home range influence the efficacy of resource exploitation [12]. If the availability of preferred cover decreases, home range size must increase to acquire more resources [13]. Given that the size of the home range may be correlated to resource phenology, the occurrence of seasonally selected covers within a matrix of marginal and/or avoided covers should have an inverse relationship with home range size [14], [15]. In addition, variability in cover diversity across a region may affect the distribution of resources and influence how animals use the landscape [16], [17]. Therefore, the diversity of selected covers and the level of heterogeneity may also share inverse relationships with home range size. Greater cover diversity and increased edge can provide multiple types of foraging and security opportunities, thereby allowing animals to use smaller areas for resource acquisition [13], [18]. Further, cover is generally arranged in patches, clusters, and close aggregations [19], such that at the extreme, relatively homogeneous areas are created [20], [21]. As a result, the configuration and distribution of important covers may require individuals to range nearer or farther in search of preferred resources and consequently influence home range size.

In this study, we tested hypotheses related to reproductive status and resource availability to explain variation in home range size for female barren-ground grizzly bears (*Ursus arctos*) of the Mackenzie Delta region in Canada’s western Arctic. We used generalized linear mixed-effects models [22] and an information-theoretic approach [23] to test competing models to explain individually-based variability in home range size. Specifically, we examined the influence of seasonal resource availability and landscape characteristics on home range size for females with and without dependent young and used resource selection analysis [24] to identify seasonally important covers. Grizzly bears living in Arctic ecosystems are small bodied, likely food-limited, and exist at low densities with some of the largest home ranges in North America [25]. Grizzly bears forage on seasonally emergent foods [26], [27], [28] and in the Mackenzie Delta, individuals have different foraging profiles from predominantly carnivorous to more herbivorous [29]. Semi-domesticated reindeer (*Rangifer tarandus tarandus*) and Arctic ground squirrels (*Urocitellus parryii*) are important high quality foods for some bears, with aquatic browsers (e.g., *Alces alces, Castor canadensis*), muskrat (*Ondatra zibethicus*), small mammals, ground-nesting birds, and fish varying in dietary frequencies depending on foraging profile [29], [30]. Herbaceous foods in the area contributing to grizzly bear diets include sweetvetch (*Hedysarum alpinum*), horsetail (*Equisetum arvense*), grass and sedges (*Carex* spp.), crowberry (*Empetrum nigrum*), buffaloberry (*Shepherdia canadensis*), bearberry (*Arctostaphylos* spp.), alpine blueberry (*Vaccinium uliginosum*), and ligonberry (*V. vitis-idaea*) [30], [31].

In addition to acquiring enough resources to maintain the individual, lactating females must also acquire enough resources to maintain milk production [5]. Where the amount and availability of quality resources is low, lactating female grizzly bears with higher nutritional needs may increase their home range size to access more food resources [2], [32], [33]. Females with small cubs-of-the-year (<12 months) must acquire enough resources for themselves and to maintain lactation but reduced mobility and behavioral adaptations to increase offspring survival may constrain home range size [4], [7]. For example, reduced movements of females with cubs-of-the-year decreases the chances of encountering infanticidal males [34]. For females with cubs ≥1 year old, increased home range size may result from the higher nutritional demands of larger offspring. Due to the energetic needs, nutritional costs associated with lactation, offspring mobility, and survival, we predicted that home range size would increase from females with cubs-of-the-year, to solitary females, followed by females with cubs ≥1 year old.

Because movement can be energetically costly, the benefits of moving must outweigh the potential risks involved in such forays. When resources, such as food, shelter or mates are abundant, animals should range over smaller areas to meet these needs [13]. Higher cover diversity results in a mosaic of available covers with more diverse resources in a smaller area, which should allow animals to move shorter distances and have smaller home ranges [18]. Based on the foraging behavior of Arctic grizzly bears, we predicted that proportional abundance of seasonally selected covers and cover diversity should share inverse relationships with home range size. In contrast, the spatial configuration of important covers on the landscape should have a positive relationship with home range size as bears use larger areas to exploit spatially patchy and dispersed resources.

## Methods

### Ethics Statement

All sampling was approved by the University of Alberta Animal Care and Use Committee for Biosciences in accordance with the Canadian Council on Animal Care guidelines (Permit numbers: ACUC412305, ACUC412405, ACUC412505, ACUC412605, and ACUC412705), and from the Government of the Northwest Territories, Department of Environment and Natural Resources, Inuvik Office (Permit numbers: WL3104, WL3122, WL3282, WL5352, and WL5375).

### Study Area

**Figure 1:**
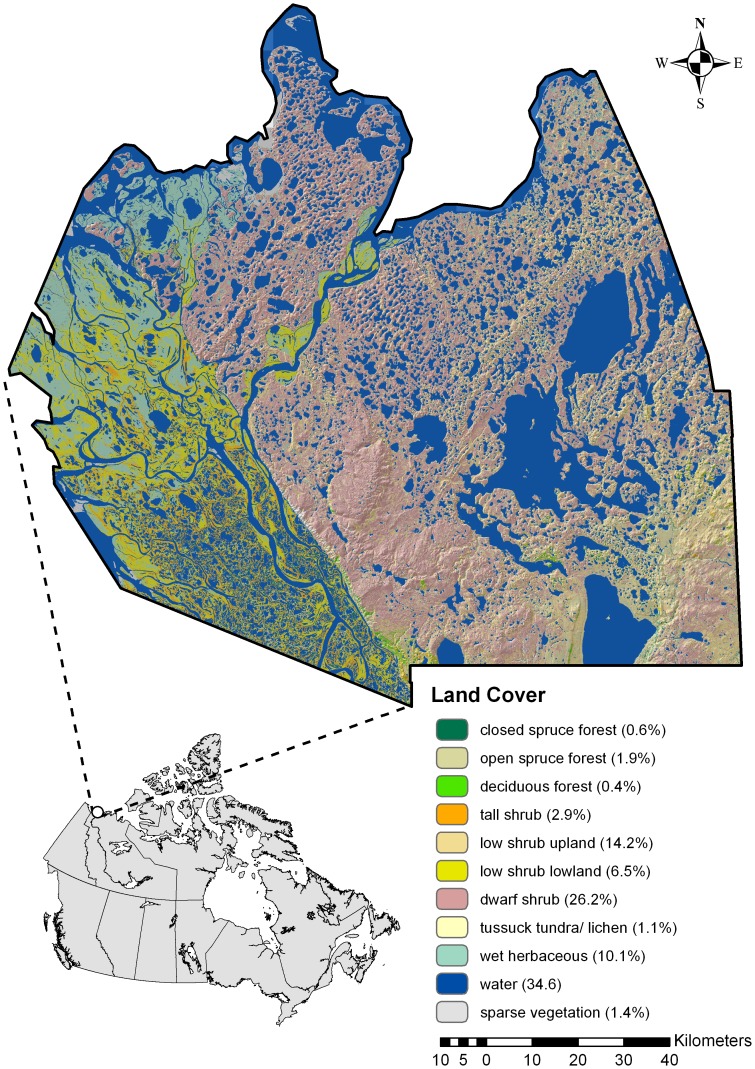
The Mackenzie Delta study area showing distributions for 11 covers, along with percentages. Lower left inset shows study area location within Canada.

This study was conducted in 2003–06 in Canada’s western Arctic near the northern edge of the North American grizzly bear’s distribution. The study area encompassed approximately 23,000 km^2^ in the Northwest Territories (Figure 1). Geographical features include numerous lakes and rivers, the Mackenzie Delta in the west, and landforms that transition south to north from boreal forest dominated by spruce (*Picea glauca* and *P. mariana*), which grade into tundra with scattered trees and shrubs, to coastal ecosystems along the Beaufort Sea [35].

Vegetation cover distribution was quantified using a decision tree [36], [37], the Landsat 5 Thematic Mapper (TM) satellite imagery classified on a 28.5-m grid, and 540 field training sites. The vegetation classification included 22 categories with a classification accuracy of ca. 90%. With little topographical relief (1–246 m) [38] and similar plant assemblages distributed widely, the area has low cover heterogeneity at the landscape scale [30], [39]. To improve model development and the interpretation, we used cover similarities to combine 22 categories into 10 major covers including 3 forest classes (open spruce forest, closed spruce forest, and deciduous forest), 4 shrub classes (dwarf shrub, low shrub upland, low shrub lowland, and tall shrub), 3 open classes (sparse vegetation, tussock tundra/lichen, and wet herbaceous), and 1 water class (Figure 1).

### Animal Capture and Monitoring

Grizzly bears were immobilized by aerial darting with tiletamine hydrochloride and zolazepam hydrochloride (Telazol®, Ayerst Laboratories Inc., Montreal, QC, Canada) at 8 mg/kg [40] shortly after den emergence in May. Approximate age was estimated at time of capture and when conditions permitted a premolar was extracted for ageing using cementum annuli [41]. Solitary bears were classified as subadults (<6 years old) or adults (≥6 years old). Reproductive status was defined by the presence or absence of dependent young (cubs-of-the-year and cubs ≥1 year old).

Bears were fitted with Gen II or Gen III: TGW-3680 Global Positioning System (GPS) (Telonics Inc., Mesa, AZ, USA) Argos satellite-linked collars (CLS America Inc., Largo, MD, USA) programmed to acquire a location once every 4 hours during the non-denning period (1 April –30 November). Collars weighed approximately 1.5 kg (<2% body weight).

### Defining Seasons

Seasonal breaks were determined using STATA 8.2 (StataCorp LP, College Station, TX, USA) and PC-ORD 5.0 [42] to perform Ward’s minimum variance cluster analysis based on changing use of the covers. We intersected locations for each bear with cover type within the geographic information system (GIS) ArcGIS 9.2 (Environmental Systems Research Institute, Redlands, CA, USA) using Hawth’s Analysis Tools for ArcGIS [43] and standardized bi-weekly cover use, which resulted in a 10-variable cluster analysis. The optimal number of seasonal-use clusters was determined using linkage distances of the dendrogram and Wishart’s [44] objective function distance and by examining the Duda and Hart index and pseudo *t* ^2^ statistic [45], [46].

### Data Analysis

#### Home range size variation

For home range estimation, grizzly bear GPS locations were analysed in ArcView 3.2 (Environmental Systems Research Institute, Redlands, CA, USA) using the Animal Movement extension [47]. We used fixed kernels to estimate 95% home range size for adult bears using least squares cross validation to estimate the smoothing bandwidth [48], [49]. Kernel home range sizes were log_10_-transformed to normalize the distribution and equalize the variance for analyses [50]. We used a one-way analysis of variance (ANOVA) followed by Tukey’s HSD test [50] to compare home range size among reproductive groups. We used a *t*-test for assessing differences in home range size between sub-adults and adults and pooled the data if appropriate. Grizzly bear home ranges in the Mackenzie Delta drift between years demonstrating low inter-annual home range site fidelity [51]. Therefore, we considered range use during each non-denning period to be independent.

#### Seasonal resource selection

To test whether home range size variation was correlated with changing cover use, we quantified selection of cover by developing seasonal selection ratios [24], [52], using GPS location data and vegetation classification. To develop selection ratios, only bears with ≥60% of their home range within the study area were included in analyses and locations outside this area were omitted. Use of different cover was estimated from GPS locations, while availability was based on random locations within each bear’s multi-annual 100% minimum convex polygon (MCP) at a sampling intensity of 1 location/km^2^ [52], [53]. Water was the reference for the categorical cover covariates because it was the most common cover with marginal importance among the bears. We used random-effects logistic regression [54], [55] in STATA to explore species-cover relationships for model development, with bear identified as the random intercept. The bear was the unit of replication, corresponding to a *design III* approach that maintained individual identity [24]. For model evaluation, we used a 80∶20 (training:testing) guideline [56] and divided location data into model-training and model-testing datasets to estimate the model and assess model accuracy, respectively.

There can be no selection where there is no variation among covers [53]. It follows that for landscapes where cover heterogeneity is low, as in our study area, resource selection models may fail to converge or have low predictive properties. Therefore, we used the Hosmer and Lemeshow approach [57] to develop seasonal resource selection models by separating those cover that were highly selected from those that were only marginally selected or avoided. Following Hosmer and Lemeshow [57] and using random-effects logistic regression, we ranked cover from most to least importance by season using the beta coefficient, Wald statistic, Wald’s *P*-value, and odds ratio. In a logistic discriminant analysis, the odds ratio is a measure of association that approximates the ratio of probabilities of the outcome given a set of predictors [57] and represents a relative selection index (RSI) (Lele S.R., unpublished data). For example, a RSI = 2.0 indicates a probability of selection twice that of other covers. We used the results of the regression analyses to create seasonal probability of selection maps using the reclassify function in the Spatial Analyst extension of ArcGIS to regroup covers into the following three categories: (1) highly selected (RSI ≥2.0), (2) matrix (marginally selected or avoided; RSI <2.0), and (3) water (reference).

We used ArcGIS to query proportions of highly selected cover and matrix units (i.e., pixels) within each bear’s multi-annual MCP. For each bear and season we estimated the proportions of used and available units of highly selected cover within the multi-annual MCP and calculated the seasonal selection ratio following Manly *et al*. [24]:
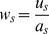
where *w_s_* is the selection ratio [24], [58] for season (*s*), *u_s_* is the proportion of used highly selected cover, and *a_s_* is the proportion of available highly selected cover. We calculated the mean selection ratio among all bears for each season and compared each mean estimate to a model where the selection ratio equalled 1 or no selection (use = available) using a Wald test in STATA. A selection ratio >1 signified that the model correctly described selection of cover in the sampled bears. Statistical significance was α = 0.05.

#### Home range size model comparison

We used the probability of selection maps that we had reclassified into highly selected, matrix, and water covers, to quantify the proportional amount of seasonally selected cover within each bear’s annual kernel home range. Proportional variables were used to account for subsequent unweighted regressions because of variation in seasonal length. We quantified the spatial structure of seasonally selected cover using Fragstats, including landscape shape index for selected cover and mean density of selected cover edge [21]. Landscape shape index is a standardized measure of the perimeter-to-area ratio, which makes it an effective means of quantifying the gradient of patches of aggregated cover to patches of highly dispersed cover [20]. We used landscape shape index as a measure of seasonally selected cover patch aggregation within each kernel home range. For example, a low landscape shape index within home ranges of varying size means that patches of selected cover are closely aggregated and a bear need not move far to access these areas and the home range should be smaller. However, if the landscape shape index is high, patches of selected cover type are highly dispersed and bears must travel farther to access these areas and the home range should be larger. Water covers 35% of the study area but provides little forage value for grizzly bears; therefore we predicted home range size to increase with water cover. We estimated the landscape shape index for water and because water creates a diversity of cover around its edges, including foraging and security cover, we used Fragstats to estimate the mean density of water edge. We also used the Shannon-Wiener index [59] to estimate overall cover diversity within the home range.

To test hypotheses to explain individually-based variability in home range size, we used a generalized linear mixed-effects model and an information-theoretic approach [22], [23]. We developed a set of candidate working models based on reproductive status, proportional amount and landscape shape index of seasonally selected cover, and landscape characteristics related to water and cover diversity. To develop the models, we used univariate analysis of each variable and Pearson’s correlation (*r*) and variance inflator function (VIF) diagnostics to identify confounding variables within each model. All variables with *r*≥|0.6|, individual VIF scores >10, or the mean of all VIF scores considerably >1 [60] were considered collinear and excluded in the same model. If two variables had a Pearson’s correlation *r*≥|0.6| the variable with the greater biological value and/or a higher significance level based on univariate analysis was included in model development.

Akaike’s Information Criterion [61] for small samples size (*AIC_c_*) and *AIC_c_* weights were used to identify the best and most parsimonious model from *a priori* candidate models that accounted for the highest explained variance [23]. We compared models for each season separately and for all seasons combined. For each season we considered that there might be an interaction between mean density of selected cover edge and landscape shape index for the selected cover. We also developed models that considered water and cover diversity independently and inclusively with the best seasonal models. Following the identification of the best and most parsimonious model, we considered polynomial forms of landscape shape indices for seasonally selected cover and water to determine if the model fit would improve. We used the *AIC_c_* weights to determine the relative importance of each variable in our best model by summing the weights of each model in the confidence set of candidate models that contained the parameter of interest (i.e., models with *AIC_c_* weights within 10% of the highest) [23], [62].

## Results

### Home Range Size

We estimated home range sizes for 29 female grizzly bears (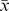 = 599 locations/bear, range = 279–959). Thirteen bears provided >1 year of data, which resulted in a total of 43 home range estimates. When the home ranges of solitary subadult (*n* = 6 ranges) and adult (*n* = 14 ranges) bears were compared we found no difference (*t*_18_ = 0.02, *P* = 0.99), therefore solitary bears were pooled. Kernel home ranges differed for females of varying reproductive status (ANOVA, *F*_2, 40_ = 4.32, *P* = 0.02). Although, home range sizes did not differ between solitary females (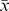 = 617 km^2^, 95% confidence interval [CI]: 422–812, *n* = 19) and females with cubs-of-the-year (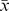 = 294 km^2^, 95% CI: 171–417, *n* = 6, Tukey’s HSD, *P* = 0.16) and females with cubs ≥1 year old (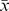 = 874 km^2^, 95% CI: 547–1201, *n* = 18, Tukey’s HSD, *P* = 0.30), the home ranges for females with cubs-of-the-year were smaller than for females with cubs ≥1 year old (Tukey’s HSD, *P* = 0.02).

### Seasonal Resource Selection

**Figure 2:**
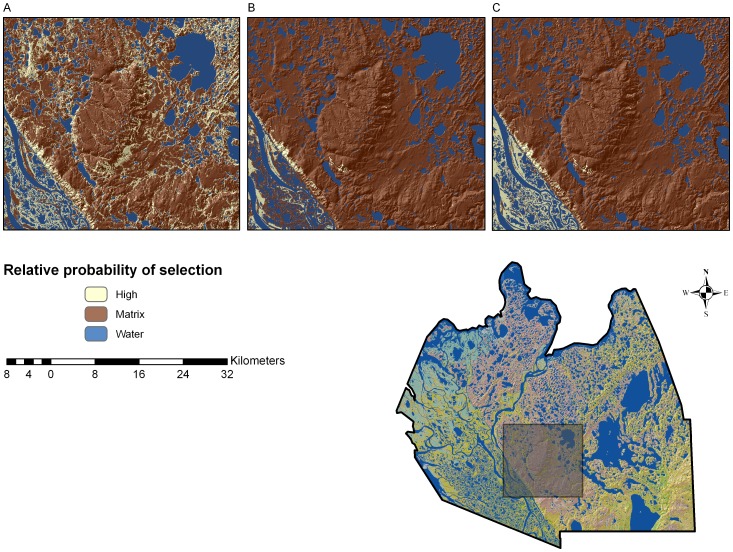
Relative probability of selection. Select portion of study area showing seasonal changes in relative probability of selection of cover for female grizzly bears monitored from 2003–06. A) den-emergence to end of week 31 (ca. 4 August, season 1); B) week 32 to the end of week 39 (ca. 29 September, season 2); and C) week 40 to the den-entry (season 3). Lower right inset shows location of highlighted portion of the Mackenzie Delta study area.

Agreement across the dendrogram, the Duda-Hart and *t* ^2^ statistic occurred at 3-clusters indicating 3 seasons [i.e., den emergence to week 31 (ca. 4 August, season 1), week 32 to 39 (ca. 29 September, season 2), and week 40 to den entry (season 3)]. For season 1, females selected sparse vegetation, tall shrub, closed spruce forest, low shrub upland, and wet herbaceous cover and were less likely to select all other covers (Table 1; Figure 2). Model validation for season 1 showed that the model described the probability of selection better than the ‘no selection model’ (*Wald test*, *F*_1,32_ = 19.22, *P*<0.001). For season 2, sparse vegetation and low shrub lowland were preferred, while all other covers were less likely to be selected (Table 2; Figure 2) and the model described the probability of selection better than the ‘no selection model’ (*F*_1, 26_ = 11.93, *P*<0.001). For season 3, selected cover included low shrub lowland, sparse vegetation, closed spruce forest, and tall shrub, while remaining covers were less likely to be selected (Table 3; Figure 2) and the descriptive ability of the model was possibly better than the ‘no selection model’ (*F*_1, 8_ = 5.02, *P* = 0.055).

**Table 1:** Univariate analysis of cover covariates for female grizzly bear season 1 [den emergence to week 31 (ca. 4 August)] resource selection analysis in the Mackenzie Delta (2003–06), Northwest Territories, Canada.

Cover	Coeff.	S.E.	z	95% CI		Wald	*P*	RSI	Use*
sparse vegetation	3.76	0.52	7.24	2.74	4.77	52.35	<0.01	42.8	h
tall shrub	1.02	0.17	5.99	0.69	1.36	35.94	<0.01	2.8	h
closed spruce forest	0.96	0.24	4.07	0.50	1.42	16.57	<0.01	2.6	h
low shrub upland	0.78	0.07	11.59	0.65	0.92	134.33	<0.01	2.2	h
wet herbaceous	0.73	0.09	8.20	0.56	0.91	67.28	<0.01	2.1	h
deciduous forest	0.51	0.25	2.02	0.01	1.00	4.08	0.04	1.7	m
open spruce forest	0.40	0.17	2.39	0.07	0.73	5.69	0.02	1.5	m
low shrub lowland	0.26	0.12	2.17	0.02	0.49	4.70	0.03	1.3	m
dwarf shrub	0.16	0.06	2.89	0.05	0.27	8.37	<0.01	1.2	m
tussock/lichen	−0.49	0.29	−1.68	−1.05	0.08	2.83	0.09	1.6	m
water	−2.37	0.10	−23.65	−2.56	−2.17	559.45	<0.01	10.7	r

* h = highly selected, m = matrix (marginally selected or avoided), r = reference.

**Table 2:** Univariate analysis of cover covariates for female grizzly bear season 2 [week 32 to 39 (ca. 29 September)] resource selection analysis in the Mackenzie Delta (2003–06), Northwest Territories, Canada.

Cover	Coeff.	S.E.	z	95% CI		Wald	*P*	RSI	Use*
sparse vegetation	3.20	0.24	13.27	2.73	3.67	176.19	<0.01	24.5	h
low shrub lowland	2.06	0.05	39.70	1.96	2.16	1575.72	<0.01	7.8	h
tall shrub	0.62	0.12	5.32	0.39	0.85	28.25	<0.01	1.9	m
low shrub upland	0.52	0.05	9.49	0.41	0.62	90.07	<0.01	1.7	m
open spruce forest	0.46	0.16	2.90	0.15	0.77	8.38	<0.01	1.6	m
wet herbaceous	0.28	0.06	4.77	0.16	0.39	22.78	<0.01	1.3	m
closed spruce forest	0.24	0.25	0.98	−0.24	0.73	0.95	0.33	1.3	m
dwarf shrub	−0.44	0.05	−9.24	−0.53	−0.34	85.43	<0.01	1.5	m
deciduous forest	−0.53	0.30	−1.75	−1.13	0.07	3.05	0.08	1.7	m
tussock/lichen	−0.69	0.24	−2.88	−1.16	−0.22	8.28	<0.01	2.0	m
water	−2.31	0.09	−26.58	−2.48	−2.14	706.45	<0.01	10.1	r

* h = highly selected, m = matrix (marginally selected or avoided), r = reference.

**Table 3:** Univariate analysis of cover covariates for female grizzly bear season 3 (week 40 to den entry) resource selection analysis in the Mackenzie Delta (2003–06), Northwest Territories, Canada.

Cover	Coeff.	S.E.	z	95% CI		Wald	*P*	RSI	Use*
low shrub lowland	2.19	0.08	26.80	2.03	2.35	718.36	<0.01	8.9	h
sparse vegetation	1.99	0.27	7.30	1.45	2.52	53.31	<0.01	7.3	h
closed spruce forest	1.49	0.27	5.58	0.97	2.02	31.10	<0.01	4.5	h
tall shrub	1.21	0.14	8.72	0.94	1.48	76.00	<0.01	3.4	h
wet herbaceous	0.33	0.10	3.36	0.14	0.52	11.31	<0.01	1.4	m
low shrub upland	0.24	0.08	3.24	0.10	0.39	10.49	<0.01	1.3	m
open spruce forest	−0.53	0.47	−1.12	−1.46	0.40	1.25	0.26	1.7	m
dwarf shrub	−0.70	0.06	−11.32	−0.82	−0.58	128.19	<0.01	2.0	m
tussock/lichen	−1.82	0.51	−3.60	−2.81	−0.83	12.95	<0.01	6.2	m
deciduous forest	−2.35	1.01	−2.32	−4.33	−0.36	5.38	0.01	10.4	m
water	−1.71	0.11	−16.14	−1.91	−1.50	260.39	<0.01	5.5	r

* h = highly selected, m = matrix (marginally selected or avoided), r = reference.

### Home Range Size Model Comparison

We found that all variables were significant correlates of home range size, with the exception of the proportional amount of season 1 selected cover and the Shannon-Wiener measure of overall cover diversity (Table 4). For seasons 1, 2, and 3 model comparisons, evaluation of Pearson’s correlation and VIF suggested that proportional amount of selected cover was correlated with the mean density of selected cover edge. For season 1, mean density of selected cover edge was a stronger correlate of home range size than proportional amount of selected cover and was therefore included in further model development (Table 4). In contrast, proportional amount of selected cover was a stronger correlate of home range size for seasons 2 and 3 than mean density of selected cover edge (Table 4). When all seasonal metrics were evaluated together, multi-colinearity among the covariates resulted in only season 1 variables available for model development and comparison, therefore no further consideration was given to the combined seasonal model. Multi-colinearity existed between landscape shape index for water and mean density of water edge. Mean density of water edge was omitted from model development because landscape shape index for water was a stronger correlate of home range size variability (Table 4).

**Table 4:** Results of 13 univariate models used for developing multivariate candidate models to explain variation in annual home range size of female grizzly bears in the Mackenzie Delta (2003–06), Northwest Territories, Canada.

Category	Variable	Mean	S.D.	*β*	S.E.	95% C.I.		*P*	*R^2^*
Reproductive status	♀+cub-of-the-year			−0.20	0.12	−0.44	0.04	0.016	0.19
	♀+cub ≥1 year old			0.16	0.10	−0.03	0.35		
	solitary ♀			2.66	0.07	2.52	2.79		
season 1	proportional amount of selected cover	0.37	0.11	0.55	0.47	−0.36	1.47	0.236	0.04
	landscape shape index for selected cover	108.42	42.32	0.01	0.00	0.01	0.01	<0.001	0.91
	mean density of selected cover edge (m^2^)	91.60	15.55	0.01	0.00	0.00	0.01	0.002	0.19
season 2	proportional amount of selected cover	0.12	0.14	−1.30	0.30	−1.89	−0.71	<0.001	0.32
	landscape shape index for selected cover	43.65	14.45	0.02	0.00	0.01	0.02	<0.001	0.34
	mean density of selected cover edge (m^2^)	20.74	19.76	−0.01	0.00	−0.01	−0.01	<0.001	0.34
season 3	proportional amount of selected cover	0.15	0.18	−1.04	−0.23	−1.49	−0.59	<0.001	0.34
	landscape shape index for selected cover	47.83	15.53	0.02	0.00	0.02	0.02	<0.001	0.63
	mean density of selected cover edge (m^2^)	23.39	19.14	−0.01	0.00	−0.01	−0.01	<0.001	0.36
water	landscape shape index for water	27.66	10.97	0.01	0.00	0.00	0.02	0.002	0.19
	mean density of water edge (m^2^)	25.25	11.98	−0.01	0.00	−0.01	0.00	0.028	0.10
cover diversity	Shannon-Wiener index (H)	1.47	0.20	−0.41	0.25	−0.90	0.09	0.106	0.07

For season 1, the best model included reproductive status and landscape shape index for season 1 selected cover, and had one competing model for the top rank, with only landscape shape index for season 1 selected cover (Table 5). The best model for season 2 included landscape shape index for season 2 selected cover and proportional amount of season 2 selected cover. The best model for season 2 also had a competitor, reproductive status, proportional amount of season 2 selected cover, and landscape shape index for season 2 selected cover. The best model for season 3 included reproductive status, proportional amount of season 3 selected cover, and landscape shape index for season 3 selected cover. For hydrology, the best model included reproductive status and landscape shape index for water. All models competing for top rank from each of the model categories (i.e., Seasons 1, 2, and 3, and hydrology) were used to create a new set of candidate models, for which *AIC_c_* and *AIC_c_* weights were recalculated for model comparison (Table 6).

**Table 5:** Candidate set of models by season and hydrology to explain variation in annual home range size for female grizzly bears monitored from 2003–06 in the Mackenzie Delta, Northwest Territories, Canada.

Category	Model description*	*k*	*AIC_c_*	Δ *AIC_c_*	*w*	*R^2^*
Season 1	rs+s1lsi	3	−74.01	0.0	0.39	0.91
	s1lsi	2	−73.68	0.3	0.33	0.91
	s1lsi+s1edge	3	−72.02	2.0	0.14	0.91
	rs+s1edge+s1lsi	4	−71.98	2.0	0.14	0.91
	rs+s1edge	3	12.79	86.8	0.00	0.32
	rs	2	16.23	90.2	0.00	0.19
	s1edge	2	17.82	91.8	0.00	0.19
	null model	1	22.28	96.3	0.00	0.00
Season 2	s2lsi+s2prop	3	−30.19	0.0	0.62	0.75
	rs+s2prop+s2lsi	4	−29.22	1.0	0.38	0.76
	rs+s2lsi	3	−2.35	27.8	0.00	0.44
	s2lsi	2	−0.94	29.3	0.00	0.34
	rs+s2prop	3	4.83	35.0	0.00	0.44
	s2prop	2	10.06	40.3	0.00	0.32
	rs	2	16.23	46.4	0.00	0.19
	null model	1	22.28	52.5	0.00	0.00
Season 3	rs+s3prop +s3lsi	4	−45.74	0.0	0.78	0.79
	s3prop+s3lsi	3	−43.22	2.5	0.22	0.78
	s3lsi	2	−28.45	17.3	0.00	0.63
	rs+s3lsi	3	−23.13	22.6	0.00	0.65
	rs+s3prop	3	1.25	47.0	0.00	0.48
	s3prop	2	8.79	54.5	0.00	0.34
	rs	2	16.23	62.0	0.00	0.19
	null model	1	22.28	68.0	0.00	0.00
hydrology	rs+wlsi	3	7.80	0.0	0.98	0.39
	rs	2	16.23	8.4	0.01	0.19
	wlsi	2	17.51	9.7	0.01	0.19
	null model	1	22.28	14.5	0.00	0.00

* rs = reproductive status, s1edge = mean density of season 1 selected cover edge, s1lsi = landscape shape index for season 1 selected cover, s2prop = proportional amount of season 2 selected cover, s2lsi = landscape shape index for season 2 selected cover, s3prop = proportional amount of season 3 selected cover, s3lsi = landscape shape index for season 3 selected cover, wlsi = landscape shape index for water.

**Table 6:** Final candidate set of model comparisons to explain variation in annual home range size for female grizzly bears monitored from 2003–06 in the Mackenzie Delta, Northwest Territories, Canada.

Model descriptions*	*k*	*AIC_c_*	Δ *AIC_c_*	*w*	*R^2^*
rs+s1lsi	3	−74.01	0.0	0.45	0.91
s1lsi	2	−73.68	0.3	0.38	0.91
rs+s1lsi+wlsi	4	−72.11	1.9	0.17	0.91
rs+s1lsi^2^	3	−60.01	14.0	0.00	0.88
rs+s3prop+s3lsi	4	−45.74	28.3	0.00	0.79
rs+s3prop +s3lsi +wlsi	5	−39.73	34.3	0.00	0.83
s2lsi+s2prop	3	−30.19	43.8	0.00	0.75
rs+s2prop+s2lsi	4	−29.22	44.8	0.00	0.76
s2lsi+s2prop +wlsi	4	−27.76	46.2	0.00	0.75
rs+wlsi	3	7.80	81.8	0.00	0.39
rs	2	16.23	90.2	0.00	0.19
null model	1	22.28	96.3	0.00	0.00

* rs = reproductive status, s1edge = mean density of season 1 selected cover edge, s1lsi = landscape shape index for season 1 selected cover, s2prop = proportional amount of season 2 selected cover, s2lsi = landscape shape index for season 2 selected cover, s3prop = proportional amount of season 3 selected cover, s3lsi = landscape shape index for season 3 selected cover, wlsi = landscape shape index for water.

We found that the best and most parsimonious model for explaining home range size variation included, reproductive status and landscape shape index for season 1 selected cover and explained 91% of the variance (Table 6):
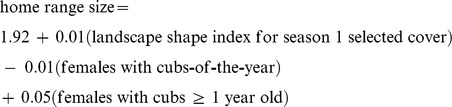



The polynomial form for landscape shape index of season 1 selected cover did not improve model fit and no significant random effect among individual bears across years was detected (*P* = 0.22). The next best model included only landscape shape index for season 1 selected cover with a Δ *AIC_c_* = 0.3, which was followed by a model with reproductive status, landscape shape index for season 1 selected cover, and landscape shape index for water with a Δ *AIC_c_* = 1.9 (Table 6). These two models had *AIC_c_* weights within 10% of the best model (*w_i_* = 0.45) [62] and Δ *AIC_c_* <2.0, and were included, along with the best model, in the confidence set of candidate models [23]. With a Δ *AIC_c_* <2.0, model-averaging may be warranted [23], however, when model-averages were estimated there was no change in the magnitude or direction of the intercept or coefficients in the model, therefore we retained our top ranking model as the best model (Table 6). Additional models had Δ *AIC_c_* values >10 (Table 6), suggesting little empirical support.

Using the *AIC_c_* weights from our confidence set of candidate models we measured the relative importance of the variables contained within our best model by estimating importance weights [23]. We found that the relative importance of both reproductive status (importance weight = 0.62) and landscape shape index for season 1 selected cover (importance weight = 1.00) resulted in both parameters being plausible explanations for home range size variation among female grizzly bears. However, given the data and the candidate models, landscape shape index for season 1 selected cover was 1.61 times more plausible than reproductive status as an explanation for variation in female home range size.

## Discussion

Although our results supported the prediction that reproductive status affects home range size of grizzly bears in the Mackenzie Delta, not all relationships between reproductive groups were as predicted. Female grizzly bears with cubs ≥1 year old had larger home ranges than that of females with cubs-of-the-year as predicted; a finding common with studies of other bear populations [32], [33]. However, in contrast to these studies and to our prediction, we observed no difference in home range size between solitary females and females with cubs ≥1 year old. Similarly, we found no difference between the home range size of solitary females and females with cubs-of-the-year. However, given that the mean home range size for solitary females (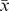 = 617 km^2^) was more than twice that of females with cubs-of-the-year (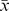 = 295 km^2^), we suggest that low sample size and the presence of a possible outlier in the dataset for females with cubs-of-the-year was the reason for a finding of non-significance between these two reproductive groups. Moreover, we found that reproductive status was of lesser importance than the individually-based cover and landscape characteristics of the area occupied in spring following den emergence into early summer (i.e., season 1). These results are consistent with the suggestion that access to high quality forage is a key resource for females with higher energy needs who require large areas to acquire necessary nutrition [2]. When bears emerge from their winter dens in spring, quality food sources are scarce and the bears are depleted of fat reserves after ca. 7 months since last eating [30]. Therefore, bears must move farther to meet their food resource needs during the early part of their active season. However, greater movement to meet these needs incurs greater risk. The search for food resources must be balanced with security, which is especially true for females with cubs-of-the-year.

The argument that home range size is influenced by an animal’s size-dependent metabolic rate [11] provides some explanation for the observed variation in home range size between females with cubs ≥1 year old and females with cubs-of-the-year. The large home range of still lactating females with larger cubs ≥1 year old is probably the result of mothers searching for quality habitat to meet the higher nutritional needs of the family group. For example, female roe deer, *Capreolus capreolus*, with fawns and newly parturient female black-faced impalas, *Aepyceros melampus petersi*, used larger ranges, which was attributed to the need to access more food to meet the increased energetic costs of lactation [63], [64]. Our results support findings that omnivore home range size increased with body mass [14]. Similarly, grizzly bear family groups in Scandinavia used large areas to meet the higher metabolic requirements of the combined body mass of the group [32].

However, in contrast to MacNab [11] and to our prediction, annual home range size did not increase from solitary females to females with cubs ≥1 year old. Studies in the central Arctic [15] and southcental Alaska [65] also found no difference in home range size between solitary females and females with cubs, although in these studies no distinction was made between females with small cubs-of-the-year versus older cubs. Because season 1 overlaps with the mating season for grizzly bears in the Mackenzie Delta, we suggest that in addition to movement to find food [2] the larger home range of solitary females may result from increased movement or roaming behavior to find mates [32]. Higher movements of females during the mating season have been reported for grizzly bears [32] and for other taxa including roe deer [66] and white-tailed deer (*Odocoileus virginianus*) [67]. Increased movement during the mating season by solitary female grizzly bears in northern Sweden was attributed to behavioral adaptations to improve opportunities for mate selection where population density is low [32]. Roaming-to-mate may reduce the occurrence of sexually-selected infanticide by increasing paternal uncertainty [7].

Although not significantly different, when we consider that the mean home range size of solitary females was twice that of females with cubs-of-the-year, which had low sample size and a possible outlier, we suggest that the reduced movements that we observed for females with cubs-of-the-year may be a counter-strategy against sexually-selected infanticide, which has been well-documented in bears [34], [68]. Sexually-selected infanticide by males is thought to improve the fitness of the perpetrator by increasing their opportunities to mate by shortening the time until a female comes into oestrous and can mate again [69]. Although it has been shown that the smaller range size of females with cubs-of-the-year is also the result of small cubs limiting the movements of their mothers [4], [70], the movements of female black bears, *Ursus americanus*, were only briefly reduced by the limited mobility of small cubs-of-the-year [71], and thus, the role of mobility and cub vulnerability remains unclear.

Variability in home range size among populations of the same species can be inversely-related to habitat productivity and food availability [72]. The large home range size for female bears in the central Arctic may be due to increased movements by bears to exploit migrating barren-ground caribou, *R. t. groenlandicus* [73], which while present in the Mackenzie Delta do not comprise a significant part of the diet [29]. In addition, although intrapopulation variability in the foraging practices of ursids has rarely been studied, and individual use of animal protein may influence home range size, no correlation as been found between home range size and foraging profiles in Mackenzie Delta grizzly bears [29].

Our results support the prediction that where preferred cover is patchy and spatially dispersed when resources are seasonally limited, bears will have larger home ranges. Conversely, proportional amount of seasonally selected cover within the home range was not found to be a strong correlate of home range size. The intrapopulation variability in home range size that we observed was largely the result of differences in the availability and spatial configuration of seasonally important cover, particularly following den emergence into early summer. For females with or without cubs, the strongest correlate of home range size was the degree of aggregation of preferred cover available during season 1 and its distribution on the landscape. Following den emergence in spring, food sources are scarce until berries ripen in late summer (i.e., season 2) and later when hibernating Arctic ground squirrels, fat with accumulated reserves, are exploited in autumn (i.e., season 3) [30]. These seasonal changes in bear foraging reflects the phenology of foods. In spring and early summer, bears selected areas consisting of tall shrub, low shrub upland, wet herbaceous, and sparse vegetation that occurs in areas along waterways and around water bodies, on steep ridges, and exposed hilltops. The covers highly selected in season 1 were also highly selected in season 3, with the exception of wet herbaceous, however the disparity between use and availability was greater in season 1. The greater variety of highly selected covers used in spring into early summer, particularly sparse vegetation, suggests increased search effort for patchy and dispersed food sources compared to more concentrated activity observed in season 2 (i.e., late summer) when berries become the dominant food [74], [75]. For bears that occupied areas where preferred cover in spring and early summer was closely aggregated, travel to access these habitats was lower, which resulted in smaller home ranges.

In contrast to our prediction, home range size was not correlated with overall cover diversity measured using the Shannon-Weiner Index. Further, season 1 selected cover edge, which is also a measure of cover diversity, did not appear in any of our candidate set of models. These results contradict the findings that increased habitat diversity results in greater resource availability within a smaller area [13], [18]. Although the greater cover diversity created by increased edge can provide many benefits, similar to a fragmented landscape, the associated matrix that separates preferred cover may require animals to move greater distances [76], [77], [78]. In highly heterogeneous areas with greater cover diversity bears may need to move farther than in more homogeneous landscapes with lower cover diversity in order to access patchy resources. Bank voles (*Myodes glareolus*) occupying fragmented and heterogeneous landscapes travelled farther to access patchy resources than those occupying non-fragmented and more homogeneous landscapes [77]. Water also contributes to the matrix separating preferred resource-rich areas. Consequently, our analysis did find some support that the spatial configuration of water was correlated with home range size variation among female reproductive groups. Where water was patchy and aggregated rather than highly dispersed across the area occupied, bears had smaller rather than larger home ranges, respectively. In areas characterised by low primary productivity, such as in deserts and Arctic environments [78], [79], long distance movements may be more pronounced as resource-rich patches are scarce, attracting individuals from long distances [78], [80]. Grizzly bears in the Mackenzie Delta exhibited low site fidelity as an adaptive foraging behavior for a region where productivity is generally low and quality habitats are spatially dispersed and temporally heterogeneous [51].

When both resource availability and population density have been considered as predictors of home range size, population density has had a greater influence than resource availability [81], [82]. Harvest of grizzly bears in the Mackenzie Delta is patchily distributed, therefore, if bear density varies across the area, a larger home range size could be related to vacancies resulting from harvest [6]. For example, removal of male adult black bears resulted in an ingress of subadult bears and an increase in population density, and a corresponding decrease in female home range size [83]. Therefore, variability in home range size among female grizzly bears may also be related to population density that we were unable to assess.

Across landscapes, cover occurs as patches, clusters and close aggregations [19], affecting the movements of animals as they search for preferred resources. To access these resources animals must range nearer or farther depending on the spatial configuration and distribution of important covers in the area occupied. It is the pursuit of these resources and the area traversed that will delineate the size of an animal’s home range [1], [2]. We have shown that for females, the presence of offspring is correlated with home range size. It is, however, the individually-based seasonal cover characteristics that had a stronger correlation with home range size. The availability of seasonally important resources in the different covers, however, remains unknown. During periods of low resource availability, even in the presence of dependent young, females travelled farther. Increase in movement to access patchy habitats during periods when resource availability is low, regardless of their spatial distribution, is common among wildlife populations [84]. However, the increased movement may increase risk to the individual or family.
